# Necrotic acalculous cholecystitis in an 8-year-old boy: a case report

**DOI:** 10.1186/s13256-025-05066-9

**Published:** 2025-02-23

**Authors:** Fanny Mueller, Amy McDonald, Vera S. Schellerer

**Affiliations:** 1 Department of Pediatric Surgery, Universitätsmedizin Greifswald, Ferdinand-Sauerbruch Straße, 17475 Greifswald, Germany; 2 Virginia Commonwealth University, College of Health Professions, 900 E Leigh St, Richmond, VA 23298 USA

**Keywords:** Pediatrics, Acute acalculous cholecystitis, Gangrenous cholecystitis

## Abstract

**Background:**

Although its incidence has increased in recent years, gallbladder inflammation in childhood is generally a rare condition. Acute acalculous cholecystitis accounts for about 50–70% of gallbladder inflammation in childhood, mostly in previously healthy children. The onset is strongly associated with viral, bacterial, or parasitic infections.

**Case presentation:**

We present the case of a healthy 8-year-old boy of German descent diagnosed with necrotic acute acalculous cholecystitis, exhibiting only mild inflammatory signs and an unspecific clinical presentation of abdominal pain. There was no evidence of viral, bacterial, or parasitic infection. According to his unclear clinical presentation with 2-day history of vomiting, we performed an explorative laparoscopy and detected a necrotic gallbladder. After laparoscopic cholecystectomy, the patient’s health status improved immediately. He was discharged from the hospital on the third day after the operation.

**Conclusion:**

Unlike our patient, almost all cases of acute acalculous cholecystitis originate from viral or bacterial infections, with clinical evidence of gallbladder inflammation shown on diagnostic imaging. An exploration of the abdominal cavity during a laparoscopic procedure was vital to our patient’s mortality and could be for others as well.

## Background

Despite the rising incidence of cholecystitis in childhood, it remains a rare event, mostly occurring in teenagers with predisposing factors such as obesity and gallstones. Patients affected below the age of 10 years are very uncommon [1]. The pathogenesis of acute acalculous cholecystitis (AAC) in childhood varies greatly, mostly being caused by systematic bacterial infections, and the majority of cases can be managed by conservative treatment [2]. In adults, gangrenous cholecystitis occurs in 30% of patients with acute cholecystitis and is a severe complication, increasing the mortality rate [3]. The gold standard of surgical treatment of cholecystitis is laparoscopic cholecystectomy; even in necrotic gallbladders, there is a low conversion rate of about 4% [4].

## Case presentation

We report on an 8-year-old boy of German descent who presented to the emergency department with acute abdominal pain. This was preceded by a general feeling of illness and intermittent vomiting for 3 days, which had stopped the day before presentation. The acute abdominal pain had started the previous night and was diffusely localized throughout the abdomen with a maximum in the right lower to middle abdomen. There had also been fecal retention for 3 days. Fever had not been present at any time. There were no relevant previous diseases, and the family history was not significant.

Physical examination revealed regular developmental status and lean nutritional status (22 kg, 129 cm). The boy was in a markedly reduced general condition with pale skin, heart rate of 102/minute, respiratory rate of 18/minute, blood pressure of 106/70 mmHg, and body temperature of 37.2 °C and appeared apathetic. There were decreased bowel sounds, tympanic palpitation, and diffuse tenderness throughout the abdomen with maximum and guarding tenderness over McBurney point and peritonism. The rest of the examination findings were normal.

Laboratory findings revealed normal leukocytes (5.41 Gpt/l [4.5–10.0]), elevated C reactive protein (33.2 mg/l [< 5.0]), elevated transaminases (ALAT: 0.58 µkatal/l [< 0.5]; ASAT: 0.71 µkatal/l [0.59]), and decreased albumin (28 g/l [34–50]). Alkaline phosphatase, bilirubin, gamma-GT, cholinesterase, creatinine, urea, and blood gas analysis were normal.

**Fig. 1:**
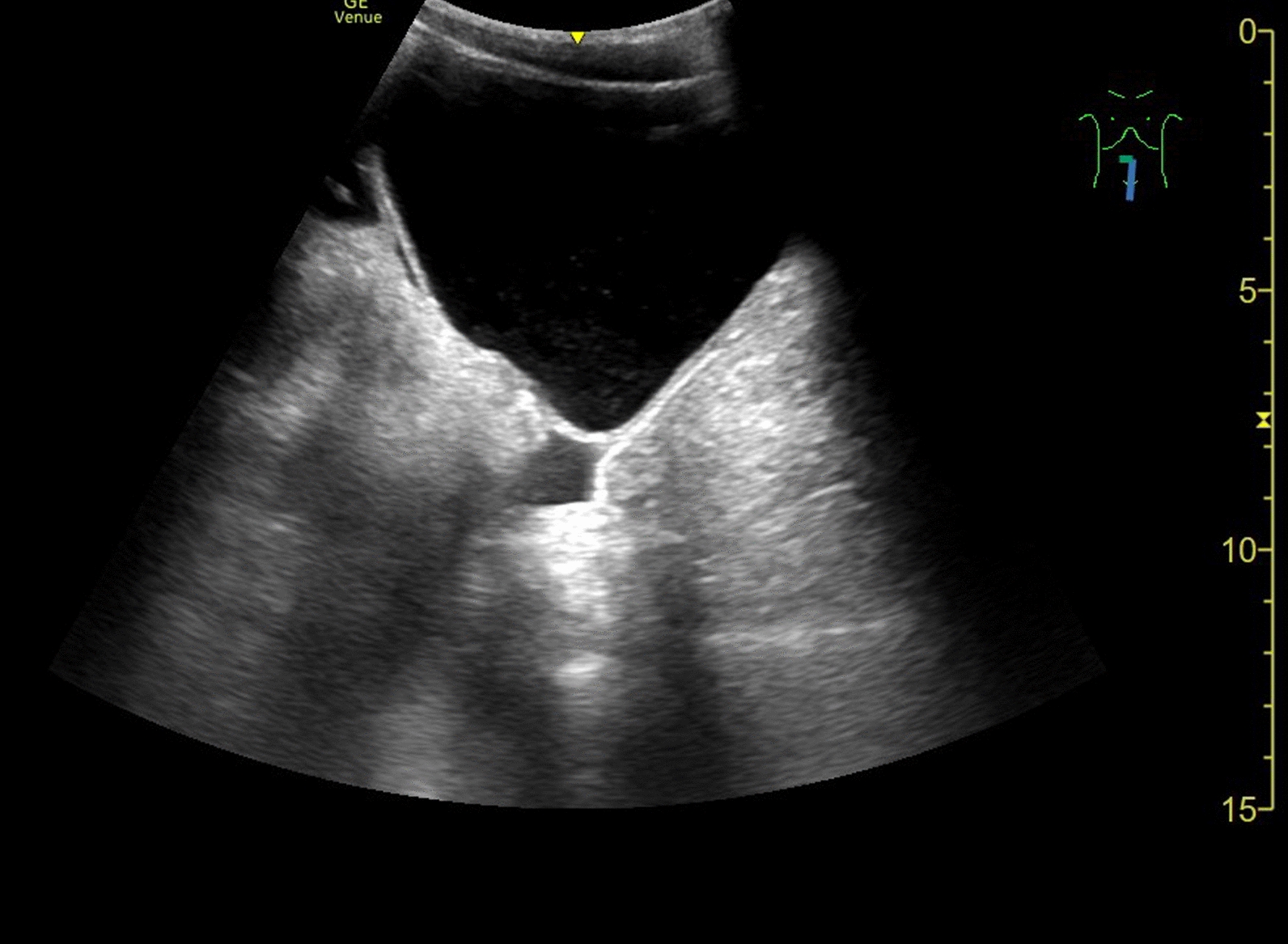
Free fluid in the lesser (true) pelvis as seen on transabdominal ultrasound

**Fig. 2:**
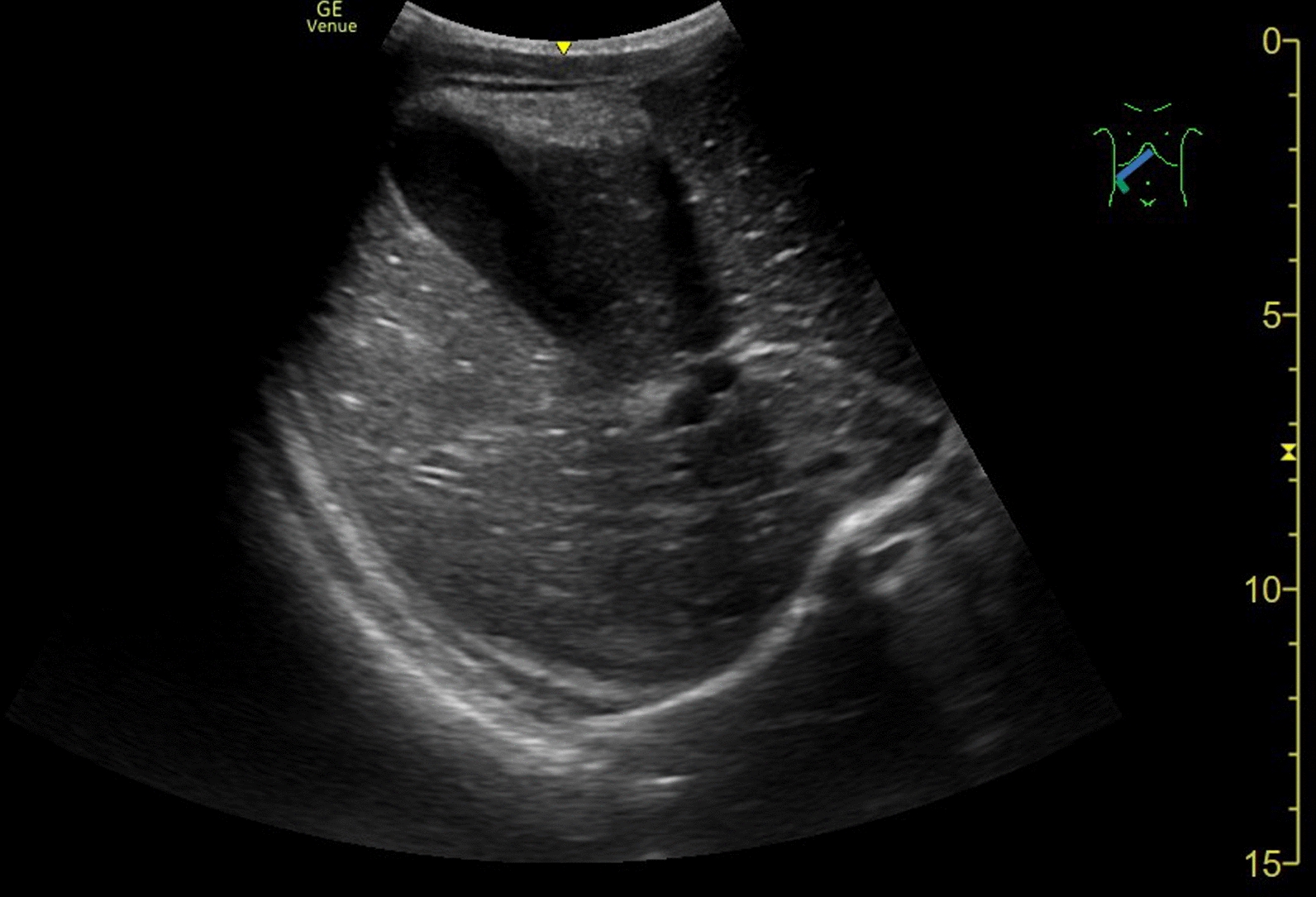
Overview of the liver and gallbladder as seen on transabdominal ultrasound

Transabdominal ultrasound showed free fluid in the lesser (true) pelvis (Fig. 1) and massive air- and stool-filled paralytic bowel loops. Owing to the artifacts, the abdominal organs were very difficult to assess but were unremarkable on overview, including the liver and gallbladder (Fig. 2). The appendix could not be visualized by ultrasound.

The sonographic findings and laboratory results did not offer clear guidance toward a specific diagnosis. In addition, the lack of significant risk factors or previous illnesses made it challenging to narrow down the list of potential differential diagnoses. As possible differential diagnoses we considered acute appendicitis, perforated Meckel’s diverticulum, ileus due to incarcerated internal hernia, and cholecystitis.

Despite laxative measures, intravenous fluid therapy, and intravenous analgesia, the patient’s condition worsened within hours of the initial presentation, with the development of guarding tenderness in the right hemiabdomen. This acute deterioration prompted a decision against further imaging and in favor of diagnostic laparoscopy to ensure adequate treatment.

**Fig. 3:**
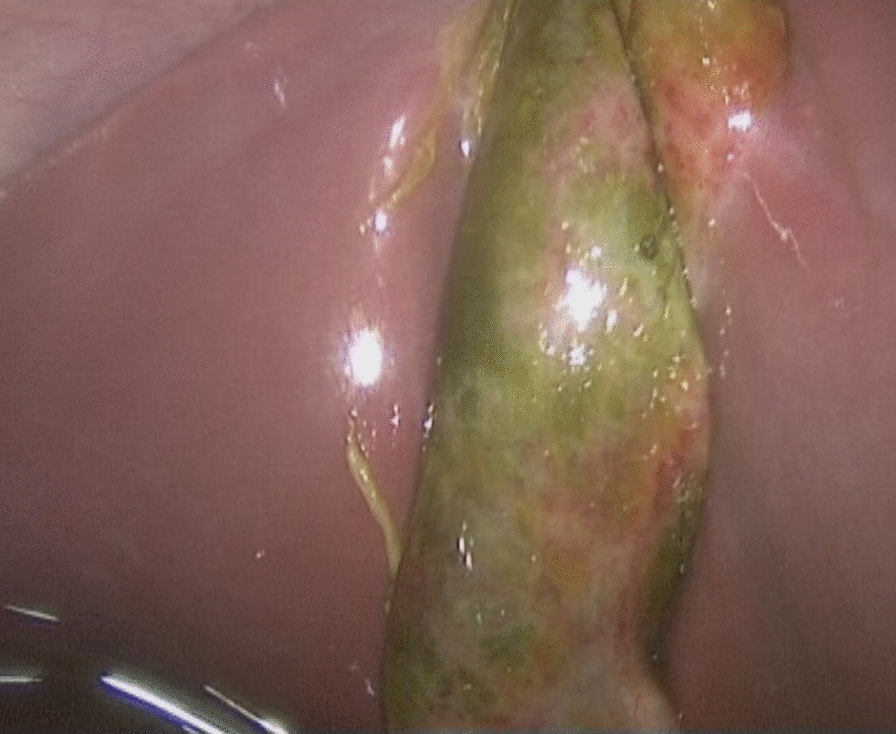
Intraoperative image showing the necrotic gallbladder

**Fig. 4:**
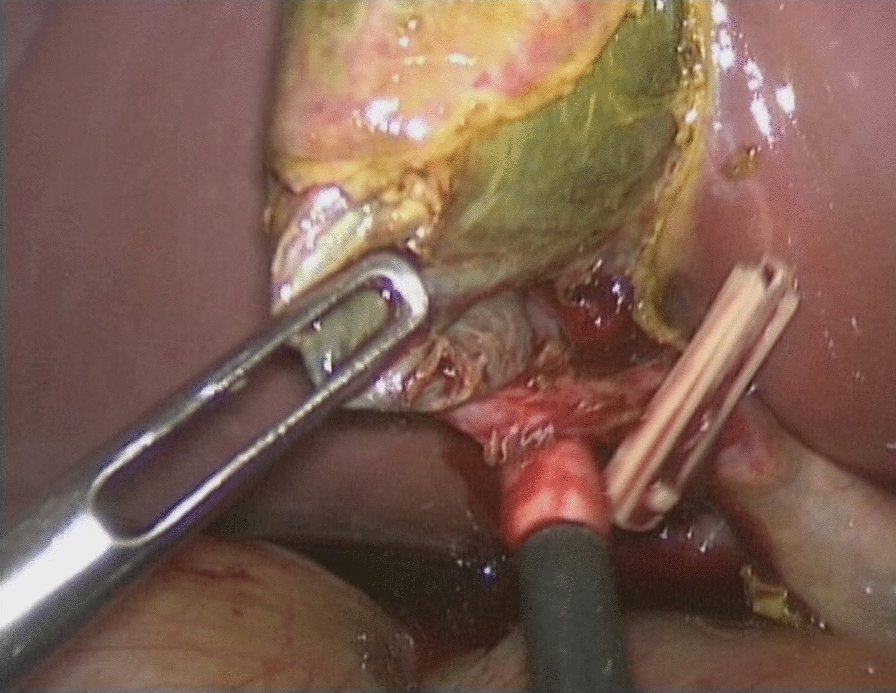
Intraoperative image of gallbladder removal using a laparoscopic clip applicator. The clip is placed on the cystic duct

As acute appendicitis was the most likely diagnosis, we started the laporoscopy with the insertion of a 10-mm trocar supraumbilically and two 5-mm trocars in the left and right lower quadrant. Intraoperatively, the appendix vermiformis presented distended and hyperemic, so appendectomy was performed. There was yellow/green clear fluid in the lesser pelvis, which was removed for microbiological examination. After appendectomy, a general exploration of the abdominal cavity was performed. There was a similar fluid around the round ligament of the liver. Therefore, we explored the gallbladder, which was fully covered by greater omentum. After removal of the inflammatory adhered fat, we detected a completely necrotic gallbladder (Fig. 3). The gallbladder was then punctured, and the resulting aspirated green bile was also saved for microbiological examination. Lastly, the gallbladder was removed by adding one further 5-mm trocar for holding the gallbladder and switching the 5-mm trocar in the lower right abdomen to a 10-mm trocar for bringing in the laparoscopic clip applicator (Fig. 4). The pathologic evaluation revealed acute acalculous gangrenous cholecystitis, pericholecystitis, and acute erosive catarrhal appendicitis.

For further diagnosis, we performed a serological examination (hepatitis A virus, cytomegalovirus, and Epstein–Barr virus), which revealed no evidence of an active infectious event. The microbiological examination of the abdominal punctate, the bile punctate, the stool, and the urine did not reveal any evidence of bacterial or parasitic infection.

Postoperatively, intravenous antibiotic therapy of cefuroxime and metronidazole was given; the symptoms resolved completely within the next 3 days.

## Discussion

Of the two categories of acute cholecystitis, 2–15% of cases consist of acute acalculous cholecystitis (AAC), inflammation of the gallbladder in the absence of gallstones. AAC is traditionally known to develop in postoperative or critically ill patients over 50 years of age [5–8]. There are also many illnesses that have been considered etiological agents. Abnormal anatomical structure of the biliary tract system, dehydration caused by burns, trauma to the gallbladder, extended parenteral nutrition, bacterial infections, viral infections, arterial hypertension, immunodeficiencies, and more have all been attributed as the possible etiologies and pathogenesis of AAC [8, 9]. Regardless of negative laboratory or imaging findings, a diagnosis of ACC can be based on right upper quadrant pain, positive Murphy’s sign, and fever [9].

Recent reports [5–8] have supplied evidence of an increased occurrence of outpatient presentations of AAC. Savoca *et al.* [5], Ryu, Ryu, and Kim [6], and Ganpathi *et al.* [8] report that the majority of patients determined to have AAC over a multiyear duration did not have a critical illness, were not postoperation, and presented de novo. Some, a minority, of the reported patients did not have risk factors.

Pediatric AAC occurrence differs greatly in many terms. Unlike adult patients, AAC is the most common form (50–70%) of gallbladder inflammation in childhood [2]. Most cases of pediatric AAC in otherwise healthy children have been attributed to viral infections (commonly HAV and EBV), while fewer have been determined to originate from bacterial infections [10, 11]. There have also been cases of AAC developing owing to blunt trauma to the abdomen, appendectomy, excessive burns, and long-term prenatal feeding, all involving comorbidities [10].

Necrotic gallbladder or gangrenous cholecystitis (GC) is a serious complication of cholecystitis, increasing the risk of morbidity [3]. As a result of prolonged ischemia, delayed treatment of acute cholecystitis increases the risk of GC [12]. Studies indicate that up to 20% of cases of acute cholecystitis develop into GC [3, 12], with delayed admission to the hospital and low leukocytes regarded as independent risk factors affecting mortality [12].

The patient we present is one of the few otherwise healthy children diagnosed with AAC that had developed to GC; unlike most others in the group, the etiology remains undetermined. Of signs and symptoms concurrent with previous cases [10, 11, 13], the patient shares abdominal pain, vomiting, nausea, abdominal tenderness, and elevated C-reactive levels, with no sign of fever, jaundice, elevated leukocytes, increased wall thickness of gallbladder, or distention of the gallbladder. Postpathological examination of the abdominal punctate, the bile punctate, the stool, and the urine did not reveal any evidence of bacterial or parasitic infection. This case underscores the diagnostic complexity of AAC in pediatric patients, especially when hallmark clinical or imaging findings are absent. Poddighe and Sazonov [2] highlight the nonspecific nature of AAC symptoms, such as abdominal pain or fever, which often mimic other intraabdominal conditions and can result in diagnostic delays. While ultrasound remains the most reliable imaging modality for AAC diagnosis [2, 10], particularly gallbladder wall thickening as the most dependable marker, no such thickening was observed in our patient, further complicating the diagnostic process. Although computed tomography (CT) offers reliable diagnostic capabilities [2], its use is limited by accessibility and concerns about radiation exposure.

Similarly, İmamoğlu *et al.* [10] emphasize that AAC diagnosis in children is challenging both clinically and in the laboratory, as its symptoms closely resemble those of other inflammatory intraabdominal diseases. The majority of cases reported by İmamoğlu *et al.* presented with abdominal pain, and tenderness, predominantly in the right upper quadrant, accompanied by leukocytosis [10]—all of which were observed in our patient. Moreover, nausea and vomiting, common in the cases described by İmamoğlu *et al.* [10], were also noted in our patient. These overlapping features further align our case with those in the literature.

İmamoğlu *et al.* [10] additionally describe distension of small bowel loops as a finding on abdominal X-rays in some cases of AAC. In our patient, a similar observation was made on ultrasound, demonstrating bowel loop distension. This highlights the importance of considering ancillary imaging findings when classic signs such as gallbladder wall thickening are absent.

A notable difference between our case and many described by İmamoğlu *et al.* is the absence of jaundice in our patient. İmamoğlu *et al.* frequently observed jaundice in AAC cases, making its absence in our patient an interesting deviation. This further illustrates the variability in AAC presentations and reinforces the need to consider the diagnosis even when certain “typical” findings are not present. İmamoğlu *et al.* [10] also emphasize that maintaining a high level of clinical suspicion is the critical first step in diagnosing AAC.

Furthermore, it is worth noting that AAC often occurs in critically ill or hospitalized children with underlying comorbidities. Therefore, the common first step in diagnosing children with AAC is recognizing prior risk factors of infectious diseases, trauma, or surgery in the patient [10]. However, in our case, the patient did not exhibit predisposing conditions such as sepsis, severe trauma, or prolonged fasting. This absence of typical risk factors underscores the need for clinicians to maintain a high degree of suspicion for AAC even in seemingly low-risk pediatric patients, particularly when laboratory and imaging findings align with broader inflammatory processes. Early recognition and targeted imaging are crucial for timely diagnosis and management.

A 2001 report by Croteau *et al.* [14] contains a case with a similar absence of predispositions and inconclusive pathological results. The 2-year-old boy, absent of relevant risk factors, presented with abdominal pain, diarrhea, fever, and reduced eating. Examination resulted with abnormal findings of increased lymphocytes, gallbladder wall thickening, and pericholecystic fluid. These last two findings do not coincide with our patient. Our patient presented with fewer clinical signs of acute necrotic gallbladder, but the most important similarity shared with our patient is the absence of typical viral and bacterial pathogens in cultures.

Given the worsening condition of the patient and symptoms indicative of a progressive intraabdominal inflammatory disease, we decided to proceed with a diagnostic laparoscopy, with appendicitis being the most likely initial diagnosis. Similarly, İmamoğlu *et al.* [10] describe cases where emergency surgical intervention, including laparotomy, was necessary to address acute abdominal conditions.

Upon laparoscopic examination of the abdomen, following the appendectomy, a short exploration of the abdomen revealed the necrotic gallbladder. Appendicitis had been assumed to cause the patient’s symptoms, so had the exploration not been performed, AAC would likely have continued to develop, eventually perforating the gallbladder wall.

## Conclusion

Despite the initial diagnostic challenges, the decision for diagnostic laparoscopy allowed for timely identification and management of the underlying pathologies, leading to the resolution of symptoms postoperatively. Therefore, we believe that, when noninvasive diagnostics reach their limits and clinical deterioration persists, invasive interventions should be considered to avoid delaying appropriate therapy. Additionally, we strongly suggest a comprehensive exploration of the entire abdominal cavity during laparoscopic procedures regardless of clinical and pathological evidence, to ensure no pathology is missed.

## Data Availability

Not applicable.

## Abbreviations

AAC: Acute acalculous cholecystitis
GC: Gangrenous cholecystitis
HAV: Hepatitis A virus
CMV: Cytomegalovirus
EBV: Epstein–Barr virus
